# On the reliability of retrieval-induced forgetting

**DOI:** 10.3389/fpsyg.2014.01343

**Published:** 2014-11-21

**Authors:** Christopher A. Rowland, Lauren E. Bates, Edward L. DeLosh

**Affiliations:** Psychology, Colorado State UniversityFort Collins, CO, USA

**Keywords:** retrieval practice, forgetting, testing, retrieval-induced forgetting, memory

## Abstract

Memory is modified through the act of retrieval. Although retrieving a target piece of information may strengthen the retrieved information itself, it may also serve to weaken retention of related information. This phenomenon, termed *retrieval-induced forgetting*, has garnered substantial interest for its implications as to why forgetting occurs. The present study attempted to replicate the seminal work by Anderson et al. (1994) on retrieval-induced forgetting, given the apparent sensitivity of the effect to certain deviations from the original paradigm developed to study the phenomenon. The study extends the conditions under which retrieval-induced forgetting has been examined by utilizing both a traditional college undergraduate sample (Experiment 1), along with a more diverse internet sample (Experiment 2). In addition, Experiment 3 details a replication attempt of retrieval-induced forgetting using Anderson and Spellman's (1995) independent cue procedure. Retrieval-induced forgetting was observed when using the traditional retrieval practice paradigm with undergraduate (Experiment 1) and internet (Experiment 2) samples, though the effect was not found when using the independent cue procedure (Experiment 3). Thus, the study can provide an indication as to the robustness of retrieval-induced forgetting to deviations from the traditional college undergraduate samples that have been used in the majority of existing research on the effect.

## Introduction

Retrieving information from memory affects the later memorability of the retrieved target itself, but also has consequences for the memorability of non-target, related information, as well. The present paper focuses on the latter. Empirical research suggests that when information is retrieved, related information can become less memorable as a consequence of the retrieval attempt. This phenomenon is known as *retrieval-induced forgetting* (RIF; Anderson et al., 1994). Investigations of RIF have had a major impact on our understanding of human memory, with a particularly strong influence on theories of forgetting.

Interference theory, one of the prominent theories of forgetting that has been developed over the last several decades, assumes that forgetting is due to retrieval failure rather than a direct weakening or loss of stored information. Retrieval failure, in turn, is thought to be a consequence of the interference that occurs when information competes for retrieval. Such views have been instantiated in detailed mathematical models, such as the Search of Associative Memory (SAM) model (Raaijmakers and Shiffrin, 1981; Mensink and Raaijmakers, 1988). In the case of SAM, information in long-term memory does not lose the strength of its representation over time, but may instead be inaccessible at a given moment, given a specific cue, when other information in memory is also strongly associated to the same cue (also see Nairne, 2002). As such, forgetting can be conceptualized as an inability to remember a target at a particular instant due to retrieval competition, competition that results in interference between a specific cue and a specific target.

Interference accounts of forgetting have been successful in explaining numerous memory phenomena (Raaijmakers and Jakab, 2013). Despite this success, Anderson and colleagues elaborated on a competing theory of forgetting that quickly gained traction. In their seminal study, Anderson et al. (1994) developed a methodology, termed the *retrieval practice paradigm*, which yielded data supportive of an inhibitory theory of forgetting. By way of comparison, interference theory suggests that information may be inaccessible when a particular cue or set of cues is present, without any impact on the stored memory itself. That is, the association between a cue and a target may be weakened or blocked, but the target, item representation remains unaffected. Inhibition theory, in contrast, suggests that forgetting can occur due to the direct weakening or suppression of information in memory. During retrieval, the representation of competing information may be suppressed, making that information less likely to be remembered in the future. Thorough reviews of interference and inhibition theories of forgetting can be found elsewhere (e.g., Anderson, 2003; Storm and Levy, 2012; Raaijmakers and Jakab, 2013), but the key point for the present purposes is that the phenomenon of retrieval-induced forgetting has sparked substantial debate in cognitive psychology regarding the nature of memory and forgetting.

Anderson et al. (1994) drew support for inhibition theory using their retrieval practice paradigm. In this paradigm, participants begin by studying lists of category exemplars (e.g., ANIMAL–CAT, ANIMAL–DOG, etc.) for a number of different categories. Following initial study, a retrieval practice phase is administered. Half of the items, selected from half of the lists initially studied, are subjected to repeated cued recall tests (e.g., ANIMAL–C___). From this manipulation, three classes of items emerge: those that are in lists which receive retrieval practice and are themselves practiced (RP+ items); those that are in lists receiving retrieval practice, but are not themselves practiced (RP− items); and those in lists that do not receive retrieval practice at all (NRP items). Typically a delay (e.g., 20 min) is administered after the retrieval practice phase, during which participants complete a distracter task. This is followed by a final memory test for the originally presented category exemplars. Retrieval-induced forgetting refers to the finding that RP− items are remembered at a lower frequency than NRP (control) items. The finding is notable as RP− and NRP items both receive identical treatment (i.e., exposure only at initial study), suggesting the differential performance at final test results from the RP− items being categorically related to those items that received retrieval practice (RP+ items). According to inhibition theory, the retrieval of RP+ items during practice has the effect of suppressing competing responses (non-target, related category exemplars, i.e., RP− items), thereby making the RP− items less accessible at final test relative to the NRP baseline items.

In contrast, interference accounts of RIF specify that any observed impairment of RP− items results not from the suppression of target representations in memory, but from interference resulting from the strengthening of category cues to RP+ items. That is, during retrieval practice, RP+ items become more strongly linked to category cues, and as such, those cues become less effective at cueing RP− items due to their strong association to RP+ items. An additional, non-inhibition based account of RIF, draws attention to the importance of context in influencing item accessibility (Jonker et al., 2013). This context-based account suggests that RIF will be observed when a shift in context occurs between initial study and retrieval practice, and additionally, when the retrieval practice context is reactivated during the final test. An NRP category as a cue at final test will reinstantiate the initial study context (as this is the only point in the retrieval practice paradigm that NRP lists are exposed), thereby allowing access to NRP items. However, given a retrieval practice category cue, the retrieval practice phase context will likely be instantiated in preference to the initial study phase (e.g., as it occurs temporally closer to the final test phase), thereby selectively facilitating access to RP+ items that were re-exposed during retrieval practice, but failing to facilitate access to RP− items that are only linked to the initial study context. Under these circumstances, internal context cues will lead to a relative bias toward sampling RP+ items, and a relative detriment to RP− items, thereby resulting in RIF. As such, both inhibitory and non-inhibitory based mechanisms have been proposed to explain the empirical observation of RIF.

The original Anderson et al. (1994) paper has received considerable attention in the literature, having been cited over 700 times, and the retrieval practice paradigm has gained widespread usage, typically with only minor deviations to the base procedure. In many cases, this has resulted in replications of the RIF phenomenon (e.g., see Storm and Levy, 2012). Notably, however, there have also been a number of failures to replicate under circumstances that closely resemble the original retrieval practice procedure, or with just minor deviations from the original methods. Moreover, given that failures to replicate an effect are rarely published (Rosenthal, 1979), it is unclear precisely how many failed (or successful) replications have been conducted and remain unreported.

RIF appears to be sensitive to a number of moderating factors. Following from the original retrieval practice paradigm (Anderson et al., 1994), RIF is commonly assessed using a category- or category plus stem–cued recall procedure. The types of cues available at final test have implications for the theoretical accounts of RIF. For example, inhibitory accounts predict that forgetting occurs due to suppression occurring at an item-specific level, and thus RIF should be observed given any final test format when assessing specific targets in memory. However, interference accounts typically only predict RIF when using final test cues that were also used during retrieval practice, and thus are not “independent” (i.e., associated with other learned items; though note there may be more subtle nuances in determining whether a given cue should be considered independent or not, e.g., Camp et al., 2007). Variations in the format of the final test have yielded inconsistent results, however. Butler et al. (2001) did not attain a RIF effect in various fragment completion tasks, with and without category cues. Rowland and DeLosh (2014) report a lack of RIF when using free recall final tests, whereas Koustaal et al. (1999) show a RIF effect in free recall. The latter authors and others (e.g., Carroll et al., 2007) failed to find RIF for final recognition tests, however, although this pattern appears to be inconsistent across the literature, as well (cf. Gomez-Ariza et al., 2005; Spitzer and Bauml, 2007). In short, variations in final test format have produced disparate results that are hard to reconcile with any single theoretical account of RIF.

RIF is also sensitive to the strength of the semantic relationship between competing targets. In accordance with the inhibition account of RIF, there must be sufficient competition between possible targets given a cue to induce suppression. Accordingly, RIF does not reliably appear with weak exemplars (Anderson et al., 1994, Experiment 2). At the other extreme, RIF may also fail to emerge if competing targets are highly similar (Shivde and Anderson, 2001; Bauml and Hartinger, 2002; Goodmon and Anderson, 2011). Thus, RIF does not seem to follow a monotonic relationship with the strength of the relationship between competing targets. Consequentially, the pattern of results expected given a specific stimuli set can be difficult to predict a priori (see Raaijmakers and Jakab, 2013, p. 103, for a related issue).

Additional failures to replicate have been reported when there were slight deviations to the original retrieval practice procedure. This has been the case when a long retention interval (e.g., 24 h) has been employed (MacLeod and Macrae, 2001; Saunders and MacLeod, 2002), when certain types of implicit final tests are used (Perfect et al., 2002), when different cues are used at final test than those employed during retrieval practice (e.g., Perfect et al., 2004; Camp et al., 2007), and when speeded responses are required during final testing (Verde and Perfect, 2011). Similarly, instructing participants to engage in an integration strategy during encoding (i.e., to intentionally relate items to one another), can yield a null RIF effect (Anderson and McCulloch, 1999; Smith and Hunt, 2000; Bauml and Hartinger, 2002), as can the use of prose materials in certain circumstances (Little et al., 2011). Furthermore, RIF may be dependent on mood state and stress level, such that inducing a negative mood (Bauml and Kuhnbandner, 2007), or high stress (Koessler et al., 2009) in participants can eliminate the RIF effect.

In a similar vein, RIF appears somewhat sensitive to the participant population used in a given study. Like much research in psychology, many RIF studies have been conducted with predominantly healthy, college enrolled participants. However, the effect appears mitigated or eliminated in clinically depressed patients (Groome and Sterkaj, 2010), and similarly, does not as consistently emerge in ADHD patients (Storm and White, 2010); populations that may have impaired inhibitory control (and thus may not be expected to show as large of a RIF effect according to inhibitory accounts of RIF). As such, although RIF has been observed in a substantial number of instances (Anderson, 2003; see Storm and Levy, 2012), it has also failed to be observed under conditions that deviate slightly from the original retrieval practice paradigm, or with changes in population.

An additional point of interest concerns demonstrations of a finding in stark contrast to RIF, termed *retrieval induced facilitation* (RIFA). Studies of RIFA utilize a paradigm highly similar, and in some cases identical to the retrieval practice paradigm. For example, Chan et al. (2006, Experiment 1) had participants study a prose passage about toucans from which two related sets of questions (Sets A and B) were derived. Some participants then engaged in retrieval practice over a subset of questions (Set A) about the passage. On a final test, performance on the previously unexposed but related questions (Set B) was facilitated by virtue of initial testing, such that Set B recall was greater than in a comparison condition where participants initially restudied (rather than retrieved) the Set A questions. Similar patterns of results have been reported in the memory literature (Chan, 2009, 2010; Cranney et al., 2009; Rowland and DeLosh, 2014), in addition to the literature on adjunct questions in educational research (in which answering questions during or after reading text materials may facilitate–rather than inhibit–the learning of related but un-tested information; see Hamaker, 1989, for a review).

Although the circumstances in which RIF fails to emerge, and similarly, in which RIF reverses to RIFA, may be viewed as boundary conditions of the RIF effect, it is important to verify and better establish the reliability and magnitude of the core RIF effect itself. Across the literature, the magnitude of RIF appears to vary widely, likely resulting from both sampling error and a variety of moderating factors. In the case of the latter, theorists have identified possible moderators that can explain some of the null RIF results in a manner consistent with inhibition theory (see, e.g., Anderson, 2003), but such explanations have also been called into question based on inconsistencies in experimental results (Raaijmakers and Jakab, 2013). Regardless of theoretical orientation, one possibility is that the RIF effect is highly sensitive to the experimental paradigm employed, such that subtle changes in method can produce large variations in results. Alternatively, the RIF effect that arises from the original RIF paradigm may be reliable but relatively small, making it difficult to detect without substantial power. Thus, a high powered replication of the original Anderson et al. (1994) study can help to establish an estimate of the effect size of RIF, whether negative, null, slight, or substantial. This, in turn, may serve as a baseline for research that seeks to specify the key variables that influence the emergence of RIF, and isolate the conditions under which RIF reverses to a facilitation effect. Some recent investigations have started to examine such issues (e.g., Chan, 2009; Little et al., 2011), and although promising, there are a number of unanswered questions given the variety of circumstances in which RIF has been failed to be observed.

In addition to replicating Anderson et al. (1994) under conditions resembling the original study (i.e., using the same methods and sampling from a similar college population), an additional replication with a more diverse population may be illuminating, given some of the individual differences that have already been identified. To this end, Experiments 1 and 2 are designed to provide highly powered replications of Anderson et al. (1994, Experiment 1). Experiment 1 was conducted as per Anderson et al. (1994), sampling from an undergraduate college population. Experiment 2 sampled from a more varied population via the internet using Amazon Mechanical Turk. Internet sampling is becoming more common in psychological research (Mason and Suri, 2011), a trend that is likely to become more prevalent, given the development and growth of tools and platforms (e.g., Amazon Mechanical Turk) that facilitate the ability of researchers to effectively and conveniently utilize internet sampling (Buhrmester et al., 2011; Mason and Suri, 2011). Internet samples tend to differ in demographic characteristics from traditional university subject pools, with internet samples being more diverse on a number of dimensions (Gosling et al., 2004; Buhrmester et al., 2011). Although an emerging literature has suggested that internet sampling procedures yield results consistent with traditional lab-based studies in certain well-established cognitive tasks (e.g., Paolacci et al., 2010), more validation is needed (Buhrmester et al., 2011). As such, a high powered RIF replication attempt with an internet sample can provide a valuable contribution to both our understanding of RIF, and more generally, the burgeoning literature on internet sampling for psychological research.

Along with two replication attempts of Anderson et al. (1994), an additional attempt to replicate a RIF effect using the independent cue procedure was conducted as Experiment 3, following Anderson and Spellman (1995, Experiment 2). The independent cue procedure is a modification to the core retrieval practice paradigm, designed in such a way to differentiate between interference and inhibitory contributions to RIF. In brief, participants follow the base retrieval practice paradigm, but at final test are cued to recall learned items by the use of novel cues, rather than cues that have established associations during the learning and retrieval practice phases of the experiment. The logic behind this method derives from the goal of attempting to disentangle potential interference and inhibitory effects from both contributing to RIF. In the standard retrieval practice paradigm, participants practice retrieval on RP+ items that belong to specific categories, and then at final test are asked to recall items from those categories using the categories themselves as cues. If RIF is observed such that RP− items (from the same categories as the RP+ items) are recalled at lower frequencies than NRP items, the increased forgetting of RP− items may reflect item-specific suppression (i.e., inhibition of RP− items as they compete for retrieval with RP+ items), but alternatively, may reflect interference. That is, strengthening the association between cues (i.e., categories) and RP+ items during retrieval practice may lessen the later likelihood of recalling RP− items because the cue to RP+ item associations interfere with ones access to the RP− items linked to the same cue (i.e., the category). In other words, retrieval practice may not lead to suppression of target information in memory, but rather could simply weaken access to certain information as a consequence of weakening the effectiveness of available cues.

The independent cue procedure from Anderson and Spellman (1995) attempts to separate interference and inhibition effects by using novel cues that have not been differentially strengthened to RP+ items during the course of the experiment. Thus, associative interference effects are presumed to be mitigated, allowing for one to interpret an observation of RIF as reflecting inhibitory processes. Experiment 3 thus attempted to replicate Anderson and Spellman (1995, Experiment 2), in order to contribute a test of cue independent RIF to the existing literature.

## Experiment 1

Experiment 1 was designed to closely replicate Anderson et al. (1994; Experiment 1). Aside from increasing the number of participants in the study, the number of stimuli learned by participants was also increased in an effort to reduce variance and maximize power.

### Method

#### Participants

An a priori power analysis using the G-Power software program (Faul et al., 2007) determined a required sampled size of 70 participants to detect a small to medium sized forgetting effect (*d* = 0.4) with 0.95 power. Observed effect size from Anderson et al. (1994) could not be computed due to insufficient data, and thus *d* = 0.4 was used as an estimate. Following from power analysis results, 70 undergraduate psychology students at Colorado State University were planned to be solicited to participate in the study. Note that with the participant session scheduling method we utilized (i.e., soliciting groups of participants at a given time), we ended up receiving data from 72 participants, and the results are presented with this full data set.

#### Design

The experiment utilized a within-participant design, manipulating item type (i.e., retrieval-practice status: RP+, RP−, and NRP, described below). Participants were randomly assigned to one of four counterbalancing conditions in which the categories presented during the practice phase were varied. The factor of retrieval-practice status has three levels, following Anderson et al. (1994): RP+ items which were exemplars belonging to a tested category that were practiced a total of three times during the initial test phase, RP− items which were exemplars belonging to a tested category that were not shown during the practice phase, and NRP items which were exemplars belonging to non-tested categories. As such, items were counterbalanced according to list type (retrieval practice lists or not), and item type within lists (RP+ or RP−).

#### Materials

***Category selection.*** Eighteen categories, two of which served as fillers, were drawn from published norms (Marshall and Cofer, 1970). Of the 18 categories, eight of them were taken directly from Appendix C of Anderson et al. (1994). The other ten categories that were created for this experiment were chosen under the same selection criteria as the original eight categories, following Anderson et al. (1994).

***Exemplar selection.*** For the eight categories obtained from Anderson et al. (1994), all strong exemplars were selected for use in this experiment. For the remaining ten categories, chosen exemplars were ensured to have an average rank order of eight according to the Battig and Montague (1969) category norms. Exemplars were low frequency, non-compound, unambiguous words with an average word frequency of 12 occurrences per million (Kucera and Francis, 1967). No two exemplars began with the same first two letters within a category, ensuring that each cue (i.e., the first two letters of each exemplar) in the practice phase was unique. In addition, the effectiveness of each cue was assessed by measuring versatility (Solso and Juel, 1980), yielding a mean versatility value of 244 (see Anderson et al., 1994, for elaboration on versatility values).

#### Procedure

The experiment consisted of four consecutive phases: a learning phase, a retrieval practice phase, a distracter phase, and a final category-cued recall phase in which the category names acted as cues for the previously studied exemplars. In the learning phase, subjects were instructed that they would be exposed to a series of word pairs containing a category and a word belonging to that category (i.e., an exemplar, e.g., Fruit: Banana), and that they would have 5 s to study each pair for a later test. After each pair had been presented for 5 s on a PowerPoint presentation, an auditory signal indicated advancement to the next word pair. The order of exemplars was determined by blocked randomization in which each block contained one exemplar from each of the 18 categories. This resulted in six blocks of 18 items each. The order within each block was randomized except for in the first block where items from the filler categories appear first, and in the last block where items from the filler categories appear last.

Next, during the retrieval practice phase, participants were shown a series of category-exemplar pair fragments with the first two letters of each exemplar as a retrieval cue (e.g., Fruit: Ba_____). Participants were instructed to write down the word with the missing letters by thinking back to the initial learning phase and recalling the exemplar that was previously shown. Ten seconds were provided per word pair, after which an auditory signal indicated that a new word pair would be shown. Category-exemplar pairs were randomly positioned, with no exemplar appearing more than once without at least one other exemplar shown in-between the two instances. Participants were exposed to each of the RP+ category-exemplar pairs three times during the practice phase. As in the learning phase, the first and last items of the practice phase belonged to the filler categories to mitigate primacy and recency effects. After completion of the retrieval practice phase, participants engaged in a 20 min operation span (OSPAN) task (Unsworth et al., 2005), serving as a distractor. The distracter OSPAN task was presented on the PowerPoint presentation and participants were instructed to record their answers on a separate answer sheet provided by an experimenter.

In the final test phase, participants were provided with the category names from the previously learned lists, and were given instructions to recall as many previously studied exemplars as possible for each category. Each of the 18 category names was presented sequentially, with 30 s given for recall of exemplars from each category before proceeding to the next category. The order of the experimental category cues was randomized, with the filler categories provided first and last (i.e., lists 1 and 18).

#### Known differences from original study

Few differences exist between our design and that of Anderson et al. (1994). However, one difference within our study was the exclusion of weak exemplars (i.e., we utilized only the “strong exemplar” condition from Anderson et al., 1994, Experiment 1), given that the strong exemplar condition produced the more robust forgetting effect the original report. One further deviation from the original study concerns the number of word lists employed. We doubled the eight experimental lists used by Anderson et al. (1994) to 16 lists (i.e., 2 filler, 16 experimental) in our study. The goal of this modification was to reduce variability and increase power.

### Confirmatory analysis plan

Our analysis plans are designed to focus on the result(s) of interest for each Experiment. For Experiment 1 we first conducted a planned comparison *t*-test between RP− and NRP item final recall performance, anticipating lower RP− performance (i.e., a RIF effect). Such a planned comparison provides the strongest means by which to detect the effect of interest. Second, we ran a one-way repeated measures ANOVA on final recall performance for all three types of items (RP+, RP−, NRP), with Bonferroni corrected *post-hoc* tests as warranted, in order to more traditionally assess the impact of the retrieval practice phase on final retention.

An additional method for evaluating replication outcomes proposed by Simonsohn (2014) centers on examining the effect sizes observed in a replication attempt against an expected minimum effect size that would be observed in the original study given an arbitrarily low amount of power. This method involves determining whether the replication attempt yields an effect that differs from the null (as per a traditional null-hypothesis significance test), in addition to an effect size based on the expected minimum effect that would be observed in the original study with 0.33 power: the “*d*_33%_ null” (refer to Simonsohn, 2014, for further elaboration on the logic and potential benefits of this method). If a replication attempt yields an effect both larger than the null, and at the same time not significantly smaller than the *d*_33%_ null, the replication is considered successful. On the other hand, replication attempts that fail to reject the null (i.e., are non-significant), but at the same time reject the *d*_33%_ null (i.e., are reliably smaller than *d*_33%_) can be considered “informative” failures to replicate. In contrast, failing to reject both the null and the *d*_33%_null provide less information, and can be considered “uninformative.” Thus, we considered the standard statistical test outcomes, along with the results of the Simonsohn (2014) method to inform the outcome of each experiment.

Participant data was only excluded if responses demonstrated an obvious lack of responding to the experimental procedure during the retrieval practice, distractor, or final recall phase (e.g., no responses given, unrelated responses given).

### Results

**Figure 1:**
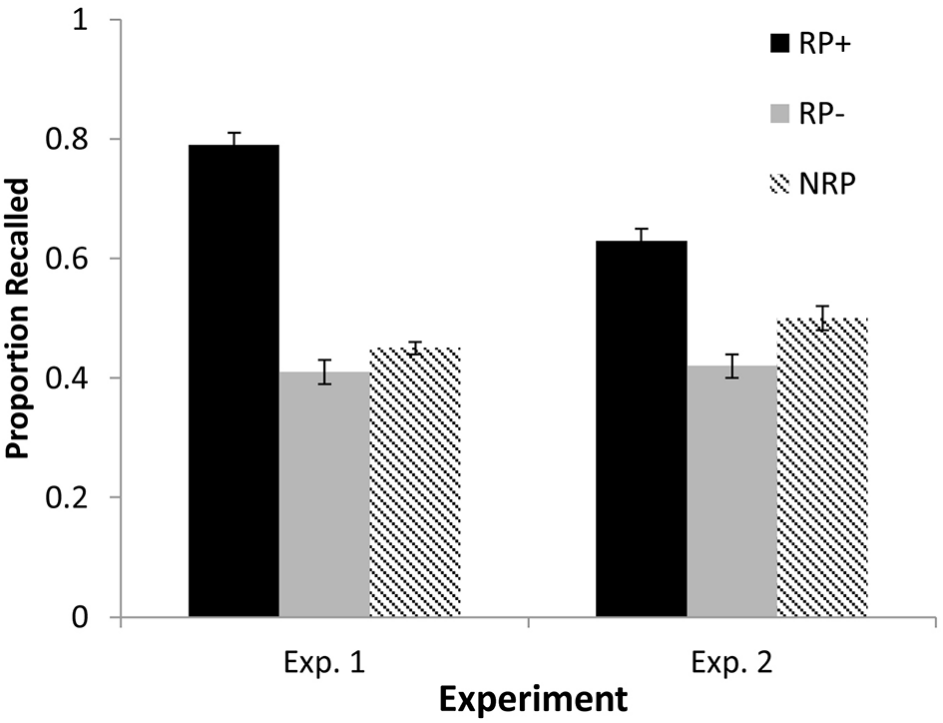
**Final recall test performance for each item type in Experiments 1 and 2**.

Final test performance is reported in Figure 1. A planned comparison examining final recall of RP− and NRP items yielded a reliable RIF effect, *t*_(71)_ = 2.59, *p* = 0.01, with NRP items recalled at a higher frequency (*M* = 0.45, *SE* = 0.01) than RP− items (*M* = 0.41, *SE* = 0.02), *d* = 0.31. A repeated measures ANOVA comparing all three item types yielded differences between final recall frequencies, *F*_(2, 142)_ = 228.87, *p* < 0.01, η^2^_*p*_ = 0.76. *Post-hoc* comparisons revealed superior recall of RP+ items (*M* = 0.79, *SE* = 0.02) to both RP− and NRP items, *p*'s < 0.01, *d*'s = 1.95 and 2.33, respectively.

We next applied the Simonsohn (2014) method to compare our RIF effect result with that of the original study. Based on the sample size from Anderson et al. (1994, Experiment 2; *n* = 36), we calculated *d*_33%_ as 0.26. Given the significant RIF effect observed in Experiment 1, and effect size *d* = 0.31, we can conclude that the replication was successful.

## Experiment 2

The goal of Experiment 2 was to provide a further replication attempt of Anderson et al. (1994; Experiment 1) through the use of a broader sample. To this end, we utilized an internet sampling procedure, as opposed to soliciting participants from our undergraduate participant pool, as was done in Experiment 1 and in the original study.

### Method

#### Participants

One-hundred and forty participants were solicited from the internet using Amazon Mechanical Turk, with the constraint that participants must indicate fluency in the English language. Participants received a small monetary compensation for their participation. Sample size was determined by doubling that of Experiment 1 to account for increased variability due to the reduced number of lists employed, as described below.

#### Materials, design, and procedure

Experiment 2 was identical to Experiment 1 with a few exceptions. First, due to the nature of online data collection, the total duration of the experiment was reduced to approximately 20 min. As such, the number of lists was reduced from 18 to four, though practice lists were not included, yielding a total of four experimental lists. Experimental lists were drawn randomly from those employed by Anderson et al. (1994). In addition, the duration of the distractor task was reduced from 20 to 5 min. The entire experiment was completed via a web browser, with responses typed into the computer. All other aspects of the procedure remained identical to Experiment 1.

#### Known differences from original study

In addition to the differences described in Experiment 1, the major deviation in Experiment 2 from Anderson et al. (1994) concerns the duration of the distractor task (5 vs. 20 min in the original study). Given that RIF appears more robust at shorter intervals (e.g., MacLeod and Macrae, 2001; Chan, 2009, 2010), we suspected that at most, the consequence of this modification would be to increase the likelihood of detecting a RIF effect. An additional deviation concerns the reduced number of lists (four, vs. eight experimental lists in the original study). In order to account for the likely increase in variability that could result from utilizing fewer lists, the sample size was increased to 140 (twice that of Experiment 1 and approximately four times that of Anderson et al., 1994).

### Confirmatory analysis plan

The plan of analysis for Experiment 2 was identical to that of Experiment 1. That is, a planned comparison of RP− and NRP item recall was conducted to examine the key effect of interest, followed by a repeated measures ANOVA and *post-hoc* tests as necessary comparing all item types. In addition, the Simonsohn (2014) method was again used to help interpret the outcome of the replication attempt. As in Experiment 1, data was only excluded from any participant who failed to respond or complete the task (e.g., due to a lost connection).

### Results

Six participants were removed from the data set and replaced due to a failure to respond during the study. Final test performance is reported in Figure 1. A planned comparison of RP− and NRP items found a reliable RIF effect, with RP− items recalled less frequently (*M* = 0.42, *SE* = 0.02) than NRP items (*M* = 0.50, *SE* = 0.02), *t*_(139)_ = 4.51, *p* < 0.01, *d* = 0.38. All item types were compared with a repeated measures ANOVA, yielding reliable differences, *F*_(2, 278)_ = 54.77, *p* < 0.01, η^2^_*p*_ = 0.28. Follow-up comparisons showed RP+ items recalled at higher frequencies (*M* = 0.63, *SE* = 0.02) than both RP− and NRP items, *p*'s < 0.01, *d*'s = 0.75 and 0.59.

As in Experiment 1, we utilized the Simonsohn (2014) replication evaluation method as a supplement to help interpret the outcome of the experiment. Again, our observed RIF effect (*d* = 0.38) was significant, and slightly larger than the *d*_33%_ from the original study (0.26). Thus, the outcome of Experiment 2 can confidently be interpreted as a successful replication.

## Experiment 3

Unlike Experiments 1 and 2, Experiment 3 attempted to replicate Anderson and Spellman's (1995, Experiment 2) method for examine RIF using an independent cue procedure. The general procedure remained similar to Experiments 1 and 2, though the modifications unique to the independent cue procedure are described.

### Method

#### Participants

As in Experiment 1, 70 participants were sampled from the participant pool at Colorado State University.

#### Materials and design

The Experiment followed that of Anderson and Spellman (1995, Experiment 2), and used materials drawn directly from the original study. Ten categories were used, six experimental and four as filler. The six experimental categories consisted of 3 pairs of related categories. Each experimental category included 6 exemplars that were unique to that category. However, three of the six exemplars for each experimental list also were common to one of three “implicit” categories. Thus, there were three pairs of experimental categories, each with six exemplars unique amongst the experimental categories, but three exemplars of which from a given list were also members of an implicit category shared by a paired experimental list. For example, drawing from Anderson and Spellman, the category “cotton” contains the exemplars “sheet” and “pajamas,” and the category “leather” contains “saddle” and “belt,” with “pajamas” and “belt” both belonging to the implicit category “clothing.” The four filler categories all contained six unique exemplars.

The experiment utilized a 2 × 4, within-participant design, manipulating item type (RP+, RP−, NRP-similar, and NRP-dissimilar) and category relatedness (related and unrelated). Half of the experimental categories were defined as retrieval practice categories, in which half of the items were actually granted retrieval practice (RP+ items), and half were not (RP− items). The other half of studied categories were not granted retrieval practice at all (NRP categories), and contained items that were either unique from all items in the experiment (NRP-Dissimilar items), or shared by implicitly related categories (NRP-Similar items). Retrieval practice and NRP categories were also defined as belonging to the related or unrelated condition based on whether the category was learned along with its paired category (i.e., where a paired category is the other member of a an experimental category pair that shares items from an implicit category).

#### Procedure

As in Experiments 1 and 2, the procedure consisted of four main phases: initial learning, retrieval practice, a distractor, and final assessment. In the initial learning phase, participants were exposed to the six exemplars from the four filler categories, along with six exemplars from four of the six experimental categories. The six items exposed for each experimental category included three unique, “dissimilar” items (i.e., items that were unique to a given category and not members of shared implicit category), and three shared, “similar” items that were explicitly members of a given category but also belonged to a shared implicit category. In total, two of the four experimental categories presented were a category pair (i.e., categories that held exemplars in a shared implicit category), along with one member of a second category pair, and one member of a third category pair. Thus, items could be considered as belong to either “related” or “unrelated” categories. Exemplars were presented in random order for 5 s each, with the specific categories assigned to the related and unrelated conditions counterbalanced across participants.

After initial learning, participants entered the retrieval practice phase where RP+ items were given three retrieval practice trials each. As in the previous experiments, each RP+ item was cued using a category and first two letters of the target (e.g., “Sharp: Ne_____”), and participants were given 10 s to retrieve the target. Retrieval trails consisted of items from the four filler categories and two experimental categories, with one of the experimental categories being from the “related” condition and one from the “unrelated” condition, as determined during initial learning. RP+ exemplars from these categories were those items assigned to be “dissimilar,” as described above. Trial order was randomized, and the categories used for retrieval practice (vs. NRP lists) were counterbalanced across participants.

A 20 min distractor task followed, after which participants were administered a final memory assessment. Category-cued recall tests for the four filler categories and four of the six experimental categories (specifically, the four experimental categories that were studied by participants during initial learning) were administered. For each of the eight category-cued recall trials, the category name was shown to participants, with instructions to write down as many items that belonged to that category that were learned during the experiment. Participants were given 30 s per category, and category order was randomized.

#### Known differences from original study

The experiment was designed to closely follow Anderson and Spellman (1995, Experiment 2). However, one difference concerned our use of a computer program to randomize and present stimuli, as described above, as an alternative to the use of generating the full set of counterbalances in the form of different answer booklets, as was done by Anderson and Spellman.

### Confirmatory analysis plan

A set of planned comparisons was conducted to assess the results of theoretical interest, following from Anderson and Spellman (1995). First, for the unrelated condition, RP+ items and corresponding NRP-Dissimilar control items were compared to assess the anticipated positive effect of retrieval practice on RP+ recall. Then, a standard RIF effect was assessed by comparing the unrelated RP− to NRP-Similar, control items. Finally, to assess cue-independent RIF, NRP-Similar items in the related condition (i.e., NRP-Similar items linked that presumably could be subject to suppression by virtue of their relation to RP− items) were compared to their control NRP-Similar items in the unrelated condition. Evaluation of the replication attempt was again supplemented by assessing the cue-independent RIF effect according to the Simonsohn (2014) method.

### Results

Three participants were removed for failure to follow task instructions, yielding 67 participants. Descriptive statistics are reported in Table 1. Analysis began by examining the effects of retrieval practice in the unrelated condition. Retrieval practice produced an expected positive effect on recall of RP+ items (*M* = 0.64, *SE* = 0.03) over NRP-Dissimilar, control items (*M* = 0.47, *SE* = 0.04), *t*_(66)_ = 4.26, *p* < 0.01. However, a RIF effect was not observed, with no reliable difference between RP− (*M* = 0.56, *SE* = 0.04), and NRP-Similar control items (*M* = 0.52, *SE* = 0.04) found, *t*_(66)_ = 0.92, *p* = 0.36. As such, the RIF results of Experiments 1 and 2 were not observed in the comparable conditions in Experiment 3. The analysis relevant to cue-independent inhibition was examined next. In particular, final recall of NRP-Similar items in the unrelated condition was compared to recall of those items in the related condition (*M* = 0.50, *SE* = 0.04), and a reliable difference was not observed, *t*_(66)_ = 0.39, *p* = 0.7.

**Table 1:** **Final recall test performance for each item type in Experiment 3**.

**Category relatedness**	**RP+**	**RP−**	**NRP-similar**	**NRP-dissimilar**
Related	0.61 (0.04)	0.39 (0.04)	0.50 (0.04)	0.41 (0.04)
Unrelated	0.64 (0.03)	0.56 (0.04)	0.52 (0.04)	0.47 (0.04)

Data reported as: Mean (SE).

We applied the Simonsohn (2014) framework to help interpret the outcome of Experiment 3. Given that the cue independent RIF effect was not significantly different from 0, we next assessed whether a 95% confidence interval around the observed effect size included the *d*_33%_ null in addition to the null of 0. Based on the sample size of Anderson and Spellman (1995, Experiment 2; *n* = 54), we computed *d*_33%_ as 0.21. The observed cue-independent RIF effect in Experiment 3 was *d* = −0.05, with a 95% confidence interval of (-0.36, 0.27). Thus, given that the effect size confidence interval includes both 0 and *d*_33%_, the replication attempt should be interpreted with caution, fitting the guidelines for an uninformative, rather than informative, failure to replicate, according to the Simonsohn (2014) guidelines. In other words, the data yielded neither an effect reliably different than 0, nor an effect reliably smaller than the minimum effect size *d*_33%_.

## General discussion

Across three experiments we attempted to replicate key findings examining retrieval-induced forgetting, using both the traditional retrieval practice paradigm (Experiments 1, 2, following Anderson et al., 1994 Experiment 1), and an independent cue procedure (Experiment 3, following Anderson and Spellman, 1995, Experiment 2). Across experiments our results were mixed, with a reliable RIF effect replicating in Experiments 1 and 2, but failing to observe in Experiment 3. We thus briefly discuss the outcomes and their implications for RIF.

The results from Experiment 1 and 2 add to a large literature showing that, with the use of the retrieval practice paradigm, retrieving information can have both positive and negative effects on memory. As anticipated, our results showed reliable and sizable positive effects of retrieval on memory for those items themselves retrieved (i.e., RP+ items, compared with NRP items), consistent with both the literature on RIF and the *testing effect* (i.e., the positive effect of retrieval on memory for those items tested, see Roediger and Karpicke, 2006; Rowland, 2014, for reviews). In addition, the empirical phenomenon of RIF was observed in both Experiments, with retrieval practice of RP+ items having a negative effect on later recall of semantically related, RP− items, when compared with NRP control items.

Although Experiment 1 utilized an undergraduate college population (similar to Anderson et al., 1994), Experiment 2 further extends RIF to a more general population, having utilized an internet sampling method. In fact, the magnitude of the RIF effect was similar in both experiments (*d*'s = 0.31 and 0.38 in Experiments 1 and 2, respectively), suggesting that RIF, as observed using the retrieval practice paradigm, may be robust across the general population. Although failures to observe RIF under certain conditions have occurred in some studies using certain clinical populations (e.g., Groome and Sterkaj, 2010; Storm and White, 2010), the present results suggest that RIF is not dependent on the use of a college population, and that failures to replicate across atypical populations may likely reflect the unique characteristics of those populations. Conversely, the present study also adds to a growing literature that internet sampling can provide a feasible means by which to study cognition, with results, in this case, conceptually consistent with those observed from an undergraduate college participant population.

The results from the Experiments 1 and 2 should be interpreted with caution as they relate to theory. Although RIF was observed in Experiments 1 and 2, note that the final test used a category cued recall procedure, such that participants could recall exemplars of a given category in any order. One drawback of this method is that such a testing protocol allows for RIF to result from output interference, and this possibility cannot be distinguished from potential inhibitory effects or other non-inhibitory mechanisms (e.g., Jonker et al., 2013; Raaijmakers and Jakab, 2013). That is, strengthened exemplars (i.e., RP+ items) may be recalled first for a given category, and subsequently interfere with the recall of other (i.e., RP−) category exemplars. Item-specific cuing procedures can resolve this issue (see Anderson, 2003), and thus may be preferred when attempting to examine theoretical characterizations of RIF.

The second major result from the present study is the failure to observe RIF in Experiment 3, which used an independent cue procedure based on that designed by Anderson and Spellman (1995, Experiment 2). The independent cue procedure was designed to assess whether RIF emerges when conditions are such that within-category interference effects are eliminated, thus allowing any observation of RIF to more uniquely reflect the operation of item-level suppression, rather than associative interference. That is, one interpretation of the data from Experiments 1 and 2 could be that participants appear to forget RP− items because the retrieval cues for those items (retrieval practice categories) become more strongly associated with the RP+ items, thus interfering with access to the RP− items themselves. By utilizing cues that are not linked to RP+ items as a way to probe memory for items that share associations with RP− items, the effects of associative interference can be mitigated, with any remaining RIF presumably resulting from item-level suppression. As such, the use of an independent cue procedure is considered to be an important test of inhibitory explanations of RIF, but has thus far yielded mixed results across the literature (e.g., for a variety of outcomes, cf., Anderson and Spellman, 1995; Williams and Zacks, 2001; Camp et al., 2007; Weller et al., 2013).

Experiment 3 did not find evidence of RIF. Aside from the possibility of a type II error, one other difference that bears mention between the present experiment and that of Anderson and Spellman (1995) concerns the sample sources. Although both experiments utilized undergraduate participant pools, the institutions differed (the University of California, Los Angeles, for Anderson and Spellman, and Colorado State University for the present experiment), and thus the participants from each study might have differed in meaningful ways that could moderate or mediate the cue-independent RIF effect. Although some work exists examining RIF with clinical populations (see Storm and Levy, 2012), little has been done to explore whether variations in cognitive traits among presumably healthy adults may interact with RIF in a systematic fashion. As such, future work may benefit from examinations of individual differences as they apply to RIF, perhaps as a way to resolve some of the apparent inconsistencies in the literature from studies using an independent cuing procedure.

Alternatively, the failure to observe RIF using an independent cuing technique can be seen as consistent with non-inhibitory based accounts of RIF. Independent cues are utilized to assess memory for targets in such a way that any forgetting observed can be attributed to a weakening of the target representation in memory, rather than a weakening of the association between a cue and target (which can result when the same, non-independent cues are used during retrieval practice and final test). The emergence of RIF using an independent cue procedure has been inconsistent across the literature (for example, cf. Storm and Levy, 2012; Jonker et al., 2013), making difficult any strong claims as to the extent that item-specific suppression may contribute to RIF, if at all. Thus, the results from Experiment 3 may be interpreted as failing to find support for an inhibitory based mechanism of RIF, with the caveat that complexities and inconsistencies across the literature preclude firm conclusions on the matter. Subsequent research will help elucidate the extent to which any given mechanism contributes to RIF given a specific set of circumstances.

In conclusion, RIF was examined across three experiments, designed to replicate the retrieval practice paradigm from Anderson et al. (1994, Experiment 1), and the independent cue procedure from Anderson and Spellman (1995, Experiment 2). Experiments 1 and 2 both observed a reliable RIF effect using the retrieval practice procedure, and the effect obtained from using both an undergraduate sample and general internet sample. However, cue independent RIF was not observed in Experiment 3. Although the observation of an empirical RIF effect reliably occurs under the original retrieval practice paradigm, the nature of the underlying mechanism(s) driving the effect remains an important question for the study of forgetting.

### Conflict of interest statement

The authors declare that the research was conducted in the absence of any commercial or financial relationships that could be construed as a potential conflict of interest.
