# Supplementary material for: On the reliability of retrieval-induced forgetting
Source: Front Psychol. 2014 Nov 21;5:1343. doi: 10.3389/fpsyg.2014.01343 (PMC4240037; doi:10.3389/fpsyg.2014.01343)
Supplement: Supplementary file 1 [file DataSheet1.DOCX]

Appendix A

Stimuli Sets

Stimuli used in Experiment 1are reported in Table A1, and for Experiment 2 in Table A2. Stimuli for Experiment 3 were drawn directly from Anderson and Spellman (1995, Experiment 2), as reported in Appendix B (p. 100) of their original publication. In Experiment 1, filler categories (see Table A1) were presented for all participants as the first and last categories studied. In Tables A1 and A2, bolded categories represent those that were blocked together for counterbalancing purposes, such that participants received either the bolded or unbolded categories as RP categories, with the other half as NRP categories. Bolded exemplars within each category represent the subset of exemplars that were treated as RP+ vs. RP- items, counterbalanced across participants. Thus, both category type (RP vs. NRP) and exemplar status (RP+ vs. RP-, for RP categories) were counterbalanced.

Table A1. *Stimuli used in Experiment 1.*

| Category | Exemplars |
| --- | --- |
| FILLER: Fuel | **Oil, Steam, Wood,** Coal**,** Electricity, Uranium |
| FILLER: Toys | **Doll, Jacks, Rattle,** Block, Car, Puzzle |
| Weapons | **Sword, Rifle, Tank,** Bomb, Pistol, Club |
| Fruits | **Tomato, Strawberry, Lemon,** Banana, Orange**,** Pineapple |
| Metals | **Nickel, Brass, Gold,** Iron, Aluminum, Silver |
| Trees | **Elm, Spruce, Hickory,** Birch, Dogwood, Redwood |
| Birds | **Crow, Duck, Sparrow,** Hawk, Woodpecker, Vulture |
| Relatives | **Cousin, Nephew, Wife,** Aunt, Husband, Son |
| Clothing | **Socks, Pants, Tie,** Jacket, Gloves, Vest |
| Diseases | **Leukemia, Smallpox, Cancer,** Measles, Flu, Cholera |
| **Drinks** | **Bourbon, Ale, Whiskey**, Vodka, Rum, Gin |
| **Fish** | **Trout,** **Bluegill, Flounder,** Catfish, Herring**,** Guppy |
| **Insects** | **Beetle, Hornet, Mosquito,** Roach, Fly, Grasshopper |
| **Professions** | **Engineer, Nurse, Plumber,** Accountant, Dentist, Farmer |
| **Time** | **Century, Week, Month,** Year, Day, Hour |
| **Furniture** | **Lamp, Dresser, Footstool,** Bed, Chair, Rug |
| **Sports** | **Hockey, Swimming, Tennis,** Wrestling, Track, Golf |
| **Weather** | **Snow, Thunder, Cyclone,** Hurricane, Lightning, Rain |

Table A2. *Stimuli used in Experiment 2.*

| Category | Exemplars |
| --- | --- |
| Fruits | **Orange, Nectarine, Pineapple,** Banana, Cantaloupe, Lemon |
| Leather | **Saddle, Gloves, Wallet**, Shoes, Belt, Purse |
| **Drinks** | **Bourbon, Scotch, Tequila,** Brandy, Gin, Rum |
| **Hobbies** | **Gardening, Coins, Stamps,** Ceramics, Biking, Drawing |
